# Antiplasmodial Activity of Nitroaromatic Compounds: Correlation with Their Reduction Potential and Inhibitory Action on *Plasmodium falciparum* Glutathione Reductase

**DOI:** 10.3390/molecules24244509

**Published:** 2019-12-10

**Authors:** Audronė Marozienė, Mindaugas Lesanavičius, Elisabeth Davioud-Charvet, Alessandro Aliverti, Philippe Grellier, Jonas Šarlauskas, Narimantas Čėnas

**Affiliations:** 1 Department of Xenobiotics Biochemistry, Institute of Biochemistry of Vilnius University, Saulėtekio 7, LT-10257 Vilnius, Lithuania; audrone.maroziene@bchi.vu.lt (A.M.); mindaugas.lesanavicius@gmail.com (M.L.); jonas.sarlauskas@bchi.vu.lt (J.Š.); 2 UMR7042 CNRS-Unistra-UHA, Laboratoire d’Innovation Moléculaire et Applications (LIMA), Bioorganic and Medicinal Chemistry Team, European School of Chemistry, Polymers and Materials, 25 rue Becquerel, F-67087 Strasbourg, France; elisabeth.davioud@unistra.fr; 3 Department of Biosciences, Universita degli Studi di Milano, via Celoria 26, I-20133 Milano, Italy; alessandro.aliverti@unimi.it; 4 MCAM, UMR7245, Museum National d’Histoire Naturelle, CNRS, 61 rue Buffon, F-75231 Paris CEDEX 05, France; philippe.grellier@mnhn.fr

**Keywords:** nitroaromatics, *Plasmodium falciparum*, ferredoxin:NADP^+^ oxidoreductase, glutathione reductase, enzyme inhibition

## Abstract

With the aim to clarify the mechanism(s) of action of nitroaromatic compounds against the malaria parasite *Plasmodium falciparum*, we examined the single-electron reduction by *P. falciparum* ferredoxin:NADP^+^ oxidoreductase (*Pf*FNR) of a series of nitrofurans and nitrobenzenes (*n* = 23), and their ability to inhibit *P. falciparum* glutathione reductase (*Pf*GR). The reactivity of nitroaromatics in *Pf*FNR-catalyzed reactions increased with their single-electron reduction midpoint potential (*E*^1^_7_). Nitroaromatic compounds acted as non- or uncompetitive inhibitors towards *Pf*GR with respect to NADPH and glutathione substrates. Using multiparameter regression analysis, we found that the in vitro activity of these compounds against *P. falciparum* strain FcB1 increased with their *E*^1^_7_ values, octanol/water distribution coefficients at pH 7.0 (log *D*), and their activity as *Pf*GR inhibitors. Our data demonstrate that both factors, the ease of reductive activation and the inhibition of *Pf*GR, are important in the antiplasmodial in vitro activity of nitroaromatics. To the best of our knowledge, this is the first quantitative demonstration of this kind of relationship. No correlation between antiplasmodial activity and ability to inhibit human erythrocyte GR was detected in tested nitroaromatics. Our data suggest that the efficacy of prooxidant antiparasitic agents may be achieved through their combined action, namely inhibition of antioxidant NADPH:disulfide reductases, and the rapid reduction by single-electron transferring dehydrogenases-electrontransferases.

## 1. Introduction

The emergence of the resistance of the malaria parasite *Plasmodium falciparum* to available drugs (e.g., chloroquine or artemisinin [1]) has resulted in the demand for new antimalarial agents and in a better understanding of their mechanisms of action. *P. falciparum* is particularly vulnerable to oxidative stress, that is, to enhanced generation of reactive oxygen species (ROS), which may be caused by the absence of the antioxidant enzymes catalase and glutathione peroxidase [2].

The antibacterial and antiparasitic activity of nitroaromatic compounds (ArNO_2_) is well known. In addition to a number of nitroheterocyclic drugs such as nifurtimox and benznidazole that have been used against Chagas disease and sleeping sickness since the 1970s, a new 5-nitroimidazole derivative, fexinidazole, has recently been approved for a treatment against sleeping sickness [3]. Frequently, the therapeutic action of ArNO_2_ is attributed to single-electron reduction into their anion radicals (ArNO_2_^−^), which in turn undergo redox cycling with the formation of ROS, or to their two/four-electron reduction into hydroxylamines (ArNHOH), able to modify DNA [4,5,6]. The single-electron reduction of ArNO_2_ is commonly performed by flavoenzymes dehydrogenases- electrontransferases, which possess natural single-electron acceptors, such as heme- or FeS-proteins [7,8,9,10]. However, there is a relative lack of information about the enzymes responsible for these reactions in parasites. Another point of view is that in trypanosomatids and *Leishmania* spp., a possible mode of ArNO_2_ action is the inhibition of the antioxidant flavoenzyme trypanothione reductase (TR) [11,12,13,14,15,16]. In this case, nitroaromatics also undergo TR-catalyzed redox cycling. In schistosomatids, a possible target of ArNO_2_ and other aromatic electron-deficient compounds is thioredoxin glutathione reductase [17,18].

A number of nitrofurans, nitrobenzenes, nitroimidazoles, and 4-nitrobenzothiadiazole were shown to possess in vitro antiplasmodial activity at micromolar or lower concentrations [19,20,21,22,23]; however, the mechanisms of their action remain poorly understood. The activity of a series of nitrobenzenes and nitrofurans roughly increased with their single-electron reduction midpoint potential (redox potential of ArNO_2_/ArNO_2_^−^ couple, *E*^1^_7_) [19], thus demonstrating a possible relationship between the compound’s ease of reductive activation and antiplasmodial activity. The antimalarial activity of nitrothiophenes was also attributed to the formation of ROS [24]. On the other hand, nitroaromatic compounds inhibit antioxidant flavoenzyme glutathione reductase from various sources [19,23,25,26]. Since *P. falciparum* glutathione reductase (*Pf*GR) plays a key role in the antioxidant defense of the parasite [2,27,28], it is believed that its inhibitors may act as efficient antiplasmodial agents. *Pf*GR is a 2 × 55 kD homodimer containing FAD and catalytic disulfide in each subunit, which catalyzes the reduction of glutathione (GSSG) at the expense of NADPH [29]. Human erythrocyte host glutathione reductase (HGR) possesses 45% amino acid sequence identity with *Pf*GR and its role in the parasite survival is a matter of debate. Both *Pf*GR and HGR are inhibited by aromatic electron-deficient compounds which were observed to bind at the dimer interface [27,28]. Our previous study demonstrated the absence of relationship between the antiplasmodial activity of nitroaromatic compounds and their efficacy as HGR inhibitors [19]. However, the relationship between the *Pf*GR inhibition and the antiplasmodial activity of nitroaromatics has not been studied so far.

Extending our previous studies [19,30], here we demonstrate that the in vitro antiplasmodial activity of nitroaromatic compounds partly correlates with their efficacy as *Pf*GR inhibitors, and partly with their reactivity with single-electron transferring *P. falciparum* ferredoxin:NADP^+^ oxidoreductase (*Pf*FNR). Given the data currently available, *Pf*FNR may act as the most efficient generator of ArNO_2_ free radicals in *Plasmodium*.

## 2. Results

### 2.1. Relationship between Antiplasmodial Activity of Nitroaromatic Compounds and Their Single-Electron Reduction Midpoint Potential

The toxicity of nitroaromatic compounds against mammalian cells and bacteria often increases with their single-electron reduction midpoint potential (redox potential of ArNO_2_/ArNO_2_^−^ couple, *E*^1^_7_). The relationship ∆log IC_50_/∆*E*^1^_7_ ~ −10 V^−1^, where IC_50_ is the compound concentration for 50% cell survival or, in the case of bacteria or parasites, for 50% growth inhibition, indicates that the main factor of cytotoxicity is redox cycling and oxidative stress [4,8,31]. Indeed, the rates of single-electron reduction of ArNO_2_ by flavoenzymes dehydrogenases-electrontransferases, such as NADPH:cytochrome P-450 reductase, ferredoxin:NADP^+^ oxidoreductase, and NO-synthase that initiate their redox cycling, increase with *E*^1^_7_ of oxidants, and are relatively insensitive to their structure [7,8,9,10].

**Figure 1:**
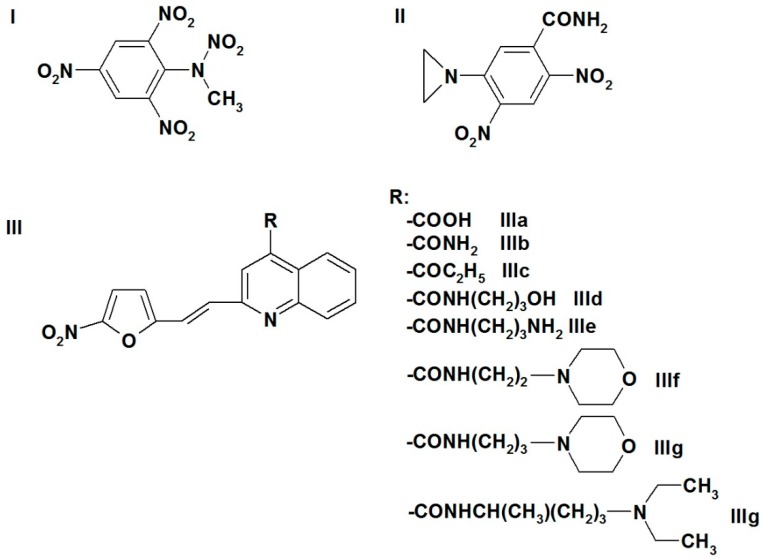
Formulae of nontrivial nitroaromatic compounds studied in this work: I, tetryl; II, CB-1954; and III, vinylquinoline-substituted nitrofurans.

In this work, we used a series of nitroaromatic compounds (Figure 1, Table 1) with available *E*^1^_7_ values. For a major part of them, the IC_50_ values against the chloroquine-resistant *P. falciparum* strain FcB1 and the inhibition efficacy against HGR were characterized in a previous work [19]. Among the examined compounds, the representatives of vinylquinoline-substituted nitrofurans (IIIa–IIIh, Figure 1) possess well-promising diverse properties such as inhibition of trypanothione reductase, that is, the potential trypanocidal activity [12] as well as bactericidal and antitumor in vitro activity [32,33], the latter property gaining increasing interest [34]. Nitrobenzenes, nitrofurantoin, and nifuroxime (compounds **1**–**9**,**12**,**14**,**23**, Table 1) were used as model compounds. Table 1 reports the IC_50_ values of compounds against *P. falciparum* strain FcB1, their *E*^1^_7_ values, and their calculated octanol/water distribution coefficients (log *D*). Importantly, the IC_50_ values for several nitroaromatic compounds obtained in separate studies were sufficiently close (Table 1). For quantitative analysis, when available, data obtained within the current work were used. The regression analysis of the log IC_50_ versus *E*^1^_7_ relationship yielded a ratio ∆log IC_50_/∆*E*^1^_7_ = −8.37 ± 1.25 V^−1^ (*r*^2^ = 0.6802) (Figure S1, Supplementary Materials). The dependence of log IC_50_ on log *D* is poorly expressed (*r*^2^ = 0.2913, Figure S2 Supplementary Materials). However, an introduction of compound log *D* as a second independent variable resulted in some improvement of the correlation:

log IC_50_ = −(0.65 ± 0.44) – (7.39 ± 1.34) *E*^1^_7_ – (0.12 ± 0.07) log *D*, (*r*^2^ = 0.7193) (1)

This shows that the oxidant potency of nitroaromatics and, to some extent, their lipophilicity play definite roles in their antiplasmodial activity.

### 2.2. Single-Electron Reduction of Nitroaromatics by *Pf*FNR and *Pf*GR

To the best of our knowledge, the pathways of reduction of nitroaromatic compounds in *P. falciparum* are not yet well understood. Among flavoenzymes dehydrogenases-electrontransferases that can initiate redox cycling of ArNO_2_, a potential candidate is ferredoxin:NADP^+^ oxidoreductase localized in the apicoplast of the parasites [36,37]. This enzyme plays a significant role in parasite survival, because the functional analysis of *P. falciparum* genome revealed a high fitness cost of disruption of its gene [38]. Table 1 lists the bimolecular reduction rate constants of ArNO_2_ by *Pf*FNR (*k*_cat_/*K*_m_). The *k*_cat_ values of the reactions were not determined because of a nearly linear dependence of reaction rate on ArNO_2_ concentration except for the most reactive oxidant tetryl (*k*_cat_ = 27.5 ± 2.0 s^−1^).

**Figure 2:**
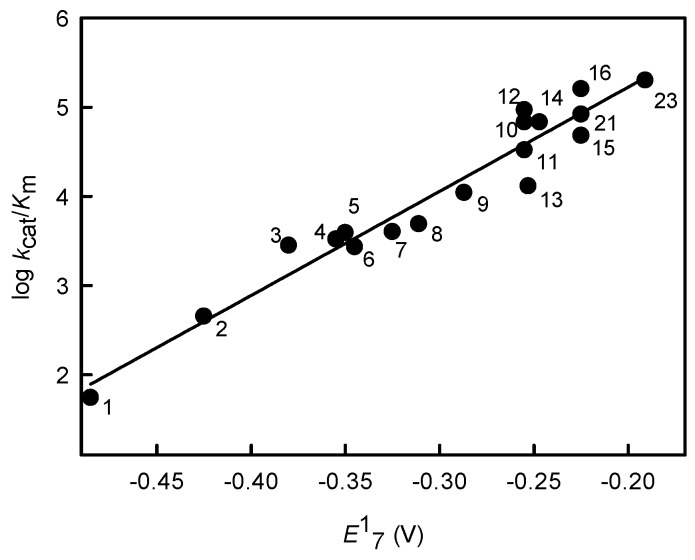
Relationship between the reactivity of nitroaromatic compounds in *Pf*FNR-catalyzed reactions (log *k*_cat_/*K*_m_) and their single-electron reduction midpoint potentials (*E*^1^_7_). The numbers of compounds correspond to those in Table 1.

**Figure 3:**
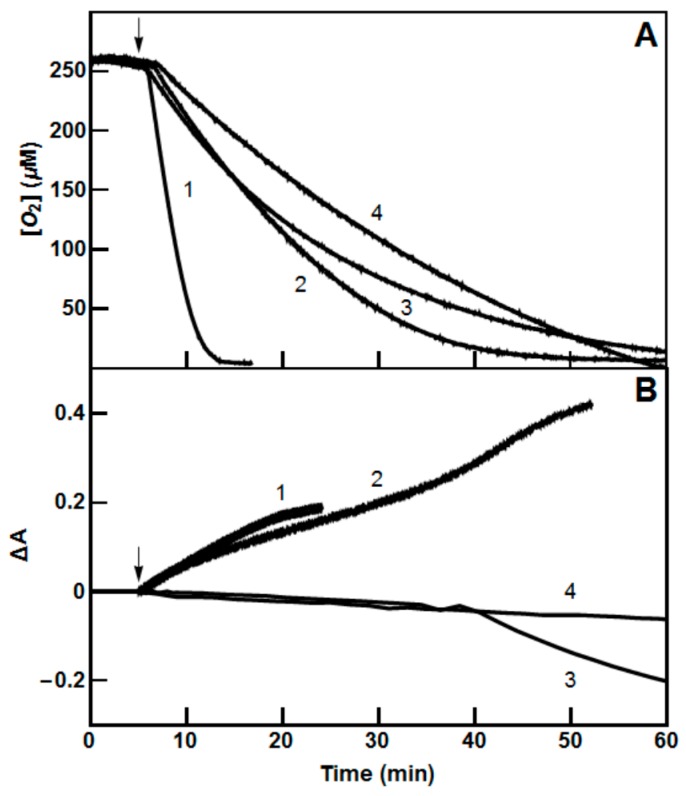
(**A**) Time course of oxygen consumption and (**B**) spectral changes during reduction of tetryl (trace 1), 1,4-dinitrobenzene (trace 2), nitrofurantoin (trace 3), and *N*-methylpicramide (trace 4) by 50 nM *Pf*FNR and NADPH-regeneration system under the absence of external oxygen supply. Compound concentration, 50 µM, absorbance monitored at 420 nm (tetryl), 340 nm (1,4-dinitrobenzene), 420 nm (nitrofurantoin), and 343 nm (*N*-methylpicramide). The arrows indicate the time of introduction of *Pf*FNR.

Figure 2 shows the linear relationship between log *k*_cat_/*K*_m_ and *E*^1^_7_ of nitroaromatics characterized by the ratio ∆log (*k*_cat_/*K*_m_)/∆*E*^1^_7_ = 11.69 ± 0.73 V^−1^ (*r*^2^ = 0.9408), which mirrors to some extent the relationship between log IC_50_ and *E*^1^_7_ (Equation (1)). *Pf*FNR catalyzes a single-electron reduction of ArNO_2_, as demonstrated by the observation that in the presence of compounds 5,12,14, and 23 (Table 1), the reduction of added cytochrome *c* takes place at rates that are 140–195% those of NADPH oxidation. Moreover, the reduction of cytochrome *c* is 15–25% inhibited by 100 U/mL superoxide dismutase. The redox cycling of ArNO_2_ is also evident from the consumption of excess O_2_ over ArNO_2_ during the reaction (Figure 3A). However, it is also important to note that *Pf*FNR-catalyzed formation of stable products of ArNO_2_ reduction does not start after complete O_2_ exhaustion, but takes place at [O_2_] = 40–50 µM after an initial lag time (in the case of *N*-methylpicramide or nitrofurantoin), or starts even without delay (in the case of tetryl or *p*-dinitrobenzene) (Figure 3B).

Both yeast and erythrocyte GR catalyze the single-electron reduction of nitroaromatics, although at a low rate [19,25]. Since the formed ArNO_2_^−^ undergoes further redox cycling, nitroaromatics are considered as “subversive substrates“ for GR. Moreover, there is some evidence that redox-active xenobiotics may be reduced at the NADP(H) binding site of GR [39,40]. It is supposed that the same reactions catalyzed by trypanothione reductase are at least partly responsible for the trypanocidal activity of nitrofurans [11]. Among the examined compounds, tetryl oxidized *Pf*GR most efficiently (*k*_cat_ = 5.9 ± 0.5 s^−1^ and *k*_cat_/*K*_m_ = 7.6 ± 0.8 × 10^3^ M^−1^·s^−1^), with parameters similar to those of HGR oxidation (*k*_cat_ ≥ 5.0 s^−1^, *k*_cat_/*K*_m_ = 2.0 × 10^3^ M^−1^·s^−1^ [37]). When present, cytochrome *c* was reduced at rates that are 170–180% those of NADPH oxidation, through a process partly inhibited by superoxide dismutase. 2,4,6-Trinitrotoluene (TNT) (*k*_cat_ = 0.2 ± 0.05 s^−1^, *k*_cat_/*K*_m_ = 150 ± 40 M^−1^·s^−1^), 1,4-dinitrobenzene (*k*_cat_ = 0.3 ± 0.07 s^−1^, *k*_cat_/*K*_m_ = 300 ± 60 M^−1^·s^−1^), and nifuroxime (*k*_cat_ ≤ 0.06 s^−1^, *k*_cat_/*K*_m_ ≤ 110 M^−1^·s^−1^) oxidized *Pf*GR much more slowly. Other nitroaromatic compounds were even less efficient oxidants of *Pf*GR. At their saturating concentrations, the rate of NADPH oxidation was almost indistinguishable from the intrinsic NADPH-oxidase activity of the enzyme, 0.07 s^−1^. This is in line with the previously reported properties of yeast and erythrocyte GR [19,25]. In conclusion, *Pf*GR-catalyzed redox cycling of nitroaromatics proceeds with much lower rates than in the analogous reaction of *Pf*FNR.

### 2.3. Inhibition of P. falciparum Glutathione Reductase by Nitroaromatic Compounds

**Figure 4:**
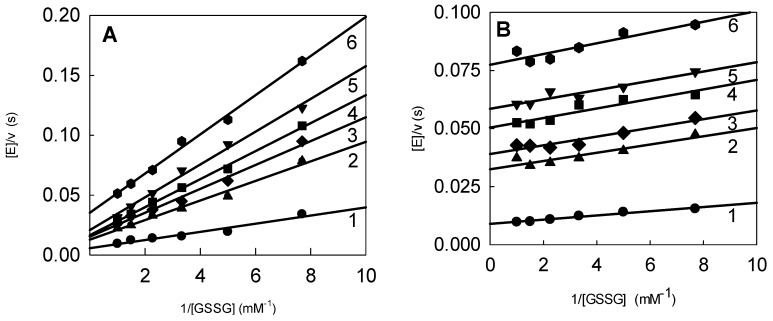
(**A**) Inhibition of *P. falciparum* glutathione reductase (*Pf*GR) by nitrofuran IIIa and by TNT (**B**) at fixed NADPH concentration, 100 µM, and varied concentrations of GSSG. (**A**) Concentrations of nitrofuran IIIa: 0.0 µM (line 1), 6.7 µM (line 2), 10.0 µM (line 3), 15.0 µM (line 4), 22.2 µM (line 5), and 50 µM (line 6). (**B**) Concentrations of TNT: 0.0 µM (line 1), 13.2 µM (line 2), 19.7 µM (line 3), 29.6 µM (line 4), 44.4 µM (line 5), and 100 µM (line 6).

**Figure 5:**
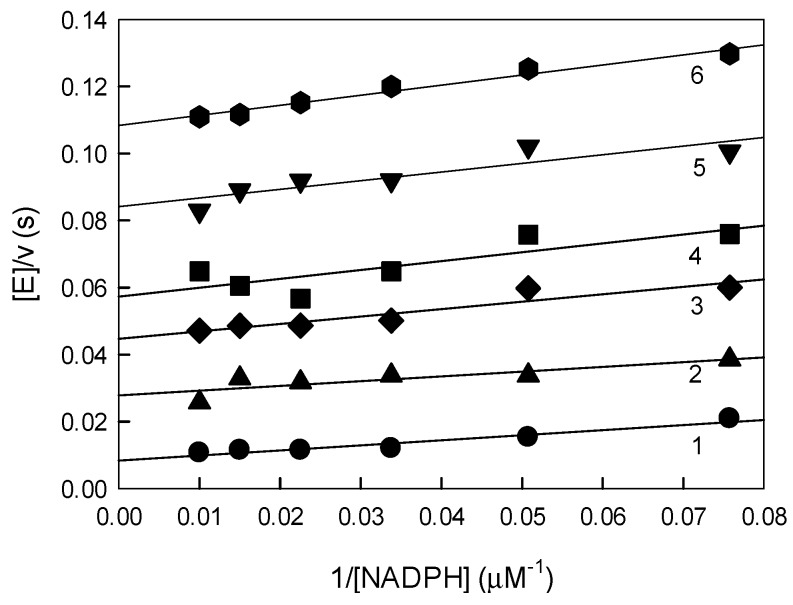
Inhibition of *Pf*GR by nitrofuran IIIa at fixed GSSG concentration, 1.0 mM, and varied concentrations of NADPH. Concentrations of inhibitor: 0.0 µM (line 1), 10.0 µM (line 2), 20.0 µM (line 3), 30.0 µM (line 4), 45.0 µM (line 5), and 66.7 µM (line 6).

Next, we analyzed the inhibition of *P. falciparum* GR by ArNO_2_. *Pf*GR acts through a “ping-pong“ reaction mechanism with separate reductive and oxidative half-reactions [29]. At saturating concentrations of substrates, 100 μM NADPH and 1.0 mM GSSG, the catalytic constant (***k***_cat_) of ***Pf***GR was 138 ± 4.0 s^−1^. Nitroaromatic compounds acted on *Pf*GR as non- or uncompetitive inhibitors with respect to GSSG at fixed NADPH concentration (Figure 4A,B). Similarly, nitroaromatics acted as uncompetitive inhibitors with respect to NADPH at fixed GSSG concentration (Figure 5).

These findings are in line with the well-characterized inhibition of yeast and human erythrocyte GR by nitroaromatics [19,25], and *Pf*GR and HGR by arylisoalloxazines and quinones [27,30,41]. The latter compounds may bind at the interface domain of two subunits of *Pf*GR in the vicinity of Val-56,56′ and Asp-58,58′, which correspond to His-75,75′ and Phe-78,78′ in HGR [27]. This site is distant from both the NADP(H)- and GSSG-binding regions. The *K*_i_ values of nitroaromatic compounds of *Pf*GR determined using GSSG as a variable substrate (Figure 4A,B), are given in Table 2. The table also contains *K*_i_ values of nitroaromatic compounds towards HGR determined previously [19] and partly within the present work. Importantly, the *K*_i_ values for several nitroaromatic compounds obtained in both studies were sufficiently close (Table 2). It should be noted that significant differences exist between HGR and *Pf*GR inhibition constants, and that their log values are poorly related (*r*^2^ = 0.3840). This may be attributed to differences in the structure, shape, and flexibility of the intersubunit regions of *Pf*GR and HGR [24,25]. However, the analysis of the inhibition profile of the two enzymes is beyond the scope of the present work.

The analysis of antiplasmodial activity of nitroaromatics (Table 1 and Table 2) shows that the dependence of log IC_50_ on log *K*_i_ is poorly expressed in the case of *Pf*GR, being characterized by *r*^2^ = 0.5878 (Figure S3, Supplementary Materials). An introduction of log *D* as a second variable improved the correlation:

log IC_50_ = (0.59 ± 0.23) + (0.61 ± 0.11) log *K*_i_ – (0.21 ± 0.07) log *D*, (*r*^2^ = 0.7253) (2)

Although the log IC_50_ of nitroaromatics also increased with their log *K*_i_ for HGR (Table 2), this dependence was poorly expressed (*r*^2^ = 0.3323). An introduction of log *D* as a second variable improved it up to *r*^2^ = 0.4543. Finally, the antiplasmodial activity of nitroaromatics was best described by a regression using *E*^1^_7_, log *K*_i_ for *Pf*GR, and log *D* as independent variables:

log IC_50_ = −(0.27 ± 0.43) – (4.16 ± 1.78) *E*^1^_7_ + (0.36 ± 0.14) log *K*_i_ – (0.15 ± 0.07) log *D*, (*r*^2^ = 0.7866) (3)

On the other hand, the use of log *K*_i_ for HGR as a variable resulted in a lower regression coefficient, and in an uncertain relationship between log IC_50_ and log *K*_i_:

log IC_50_ = −(0.70 ± 0.46) – (8.24 ± 1.90) *E*^1^_7_ − (0.10 ± 0.15) log *K*_i_ – (0.13 ± 0.08) log *D*, (*r*^2^ = 0.7252) (4)

## 3. Discussion

Our study resolves the debated problem about the mechanisms of antiplasmodial activity of nitroaromatics [19,23,26], and demonstrates that their activity increases both with the ease of their bioreductive activation, expressed as the value of *E*^1^_7_, and the efficiency of inhibition of *Pf*GR (Equation (3)). These data complement our previous findings on the role of inhibition of *Pf*GR in the activity of quinones against the same strain [30]. In both cases, the relationship between the activity of compounds and their efficiency as *Pf*GR inhibitors is revealed using multiparameter regression analysis. On the analogy with Equation (3), the activity of quinones was described by the relationships with ∆log IC_50_/∆log *K*_i_ = 0.633 – 0.763 [30]. These observations point to the importance of *Pf*GR as potential target for antiplasmodial agents. Apart from protection against the oxidative stress, *Pf*GR supplies GSH for the glyoxalase system, for the degradation of uncrystallized ferriprotoporphyrin IX, and as a source of reducing equivalents for ribonucleotide synthesis and thioredoxin-dependent antioxidant system [43,44,45,46]. Although no *Pf*GR knockout data are available in the case of *P. falciparum*, the functional analysis of its genome revealed a high fitness cost of disruption of the *Pf*GR gene [38]. On the other hand, the comparison between Equations (3) and (4) points to an insignificant role of HGR inhibition in the antiplasmodial action of nitroaromatics. Although the role of HGR in the survival of *P. falciparum* is a matter of debate [2,47], our data are in favor of its minor importance for parasite killing. However, this does not discard its role in the protection against an oxidative environment which limits the parasite infection rate, as it is in the case of glucose-6-phospate dehydrogenase deficiency [40]. In this context, one may note that the relationship ∆log IC_50_/∆log *K*_i_ (Equation (3)) is significantly lower than unity. It may point to a limited role of inhibition of *Pf*GR in the antiplasmodial activity of nitroaromatics. On the other hand, the disturbance of GSSG/GSH homeostasis under the oxidative or alkylative stress in certain cases may enhance the expression of GR as a compensation mechanism. This phenomenon has been observed in yeast, plants, and mammalian cells [48,49,50,51].

In this context, two other issues related to the mechanism of antiplasmodial activity of ArNO_2_ may be discussed. First is the dependence of their IC_50_ values on their *E*^1^_7_ (Equation (1)), the latter being associated with their redox cycling activity. However, because *P. falciparum*, during its intraerythrocyte stage, adopts microaerophilic metabolism and relies mainly on anaerobic processes [52,53], the role of the ROS-promoted parasite death should be interpreted with caution. On the other hand, Equation (1) may equally well reflect the rates of formation of DNA-damaging hydroxylamines under the action of single-electron transferring enzymes such as *Pf*FNR (Figure 3A,B) which may take place under partly anaerobic conditions. Another point is the role of *Pf*FNR in the formation of free radicals and/or other reduced forms of ArNO_2_ in plasmodia. To the best of our knowledge, nitroreductase activity of *P. falciparum* flavoenzymes has not been previously evaluated. Some conclusions may be drawn from the enzyme reactivity with a model compound menadione (2-methyl-1,4-naphthoquinone, *E*^1^_7_ = −0.20 V) (Table 3), because, as a rule, flavoenzymes reduce quinones much faster than nitroaromatics, or, in exceptional cases, with similar rates ([7,53], and references therein). Thus, given the data currently available (Table 3), *Pf*FNR may act as the most efficient generator of ArNO_2_ free radicals in plasmodia. *P. falciparum* thioredoxin reductase may be next to it according to menadione reductase activity (Table 3). On the other hand, the nitroreductase activity of mitochondrial type II NADH dehydrogenase is expected to be very low because menadione is slowly reducible. To the best of our knowledge, nitroaromatic compounds and soluble quinones have not been previously studied as oxidants of other enzymes of the mitochondrial respiratory chain, namely dihydroorotate dehydrogenase, succinate dehydrogenase, and malate:quinone oxidoreductase.

A more general conclusion following from our study is that the efficacy of redox active antiparasitic agents such as quinones, nitroaromatic compounds, isoalloxazines, and aromatic *N*-oxides [6] may be achieved through two separate types of action, namely inhibition of antioxidant NADPH:disulfide reductases and rapid reduction by flavoenzymes dehydrogenases- electrontransferases. Currently, these groups of compounds are mainly considered as “subversive substrates” of disulfide reductases such as glutathione reductase, trypanothione reductase, or thioredoxin reductase [11,30,40,54,56]. However, the single-electron reduction of the above compounds by dehydrogenases-electrontransferases of different origin (e.g., mammalian NADPH: cytochrome P-450 reductase, NO-synthase, NADH:ubiquinone reductase, algal FNR, and bacterial flavohemoglobin) is usually faster [7,8,9,10]. Thus, more attention should be given to the studies of these reactions catalyzed by parasite enzymes of this group.

## 4. Materials and Methods

### 4.1. Materials

Recombinant ***Pf***GR and *P. falciparum* ferredoxin:NADP^+^ oxidoreductase were prepared as previously described [29,36], and their concentrations were determined spectrophotometrically according to ***ε***_461_ = 11.7 mM^−1^·cm^−1^ and ***ε***_461_ = 10.1 mM^−1^ cm^−1^, respectively. Recombinant HGR was obtained from Sigma-Aldrich (St. Louis, MO, USA), and its concentration was determined according to ε_464_ = 11 mM^−1^·cm^−1^.

Nitrobenzene derivatives **1**,**2**,**4**–**9**,**12** and nitrofurans **10**,**11** (Table 1) were obtained from Sigma-Aldrich (St. Louis, MO, USA) and used as received. TNT, 2,4,6-trinitrophenyl-*N*- methylnitramine (tetryl) (Figure 1), and *N*-methylpicramide were synthesized as described in [57,58]. 5-(Aziridin-1-yl)-2,4-dinitrobenzamide (CB-1954, Figure 1), synthesized as described in [59], was a generous gift of Dr. Vanda Miškinienė (Institute of Biochemistry, Vilnius, Lithuania). Vinylquinoline-substituted nitrofurans IIIa–h (Figure 1) were synthesized as described in [60,61]. All synthesized compounds were previously verified by determining their melting point, as well as their ^1^H-NMR, UV, and IR spectra [8,19,25]. The purity of compounds, determined using a high-performance liquid chromatography system equipped with a mass spectrometer (LCMS-2020, Shimadzu, Kyoto, Japan), was >98%. Cytochrome ***c***, NADPH, GSSG, glucose-6-phosphate, glucose-6-phospate dehydrogenase, superoxide dismutase, and other compounds were obtained from Sigma-Aldrich (St. Louis, MO, USA) and used as received.

### 4.2. Methods

#### 4.2.1. Enzyme Kinetic Studies

All kinetic experiments were carried out spectrophotometrically using a PerkinElmer Lambda 25 UV–VIS spectrophotometer (PerkinElmer, Waltham, MA, USA) in 0.1 M K-phosphate buffer (pH 7.0) containing 1 mM EDTA at 25 °C. The steady-state parameters of reactions, the catalytic constants (*k*_cat(app.)_), and the bimolecular rate constants (or catalytic efficiency constants, *k*_cat_/*K*_m_) of the oxidants at fixed concentrations of NADPH correspond to the reciprocal intercepts and slopes of Lineweaver–Burk plots, [E]/*v* vs. 1/[oxidant], where *v* is the reaction rate, and [E] is the enzyme concentration. *k*_cat_ represents the number of molecules of NADPH oxidized by a single active center of the enzyme per second. The rates of *Pf*FNR- and *Pf*GR-catalyzed NADPH oxidation in the presence of nitroaromatic compounds or GSSG were determined using the value Δ***ε***_340_ = 6.2 mM^−1^·cm^−1^. The rates were corrected for the intrinsic NADPH-oxidase activity of enzymes, which were equal to 0.12 s^−1^ and 0.07 s^−1^ for *Pf*FNR and *Pf*GR, respectively. In separate experiments, in which 50 µM cytochrome ***c*** were included in the reaction mixture, its nitroaromatic-mediated reduction was measured using the value Δ***ε***_550_ = 20 mM^−1^·cm^−1^. The kinetic parameters were obtained by the fitting of kinetic data to the parabolic expression using SigmaPlot 2000 version 11.0 (https://systatsoftware.com). The rates of reduction of nitroaromatic compounds (50 μM) by *Pf*FNR in the absence of external oxygen supply were monitored in the presence of an NADPH-regeneration system (50 μM NADPH, 10 mM glucose-6-phosphate, and 50 U/mL yeast glucose-6-phosphate dehydrogenase) at the specific ***λ***_max_ of absorbance of compounds. In these cases, a sealed spectrophotometer cell was completely filled by the solution containing nitroaromatic compound and NADPH-regeneration system, and the reaction was initiated by the injection of *Pf*FNR. In parallel, the rate of oxygen consumption was monitored under identical conditions using a Digital Model 10 Clark electrode (Rank Brothers Ltd., Bottisham, UK). In reversible inhibition studies of *Pf*GR, reaction rates were determined either at fixed NADPH concentration (100 μM) and varied GSSG concentrations (1.0–0.13 mM), or at fixed GSSG concentration (1.0 mM) and varied NADPH concentrations (8–50 μM), and either in the absence or presence of the inhibitor at 4–6 different concentrations. Using tetryl as inhibitor, the reaction rates were corrected for *Pf*GR- catalyzed NADPH oxidation by tetryl, which was typically less than 0.4% of total reaction rate. Since CB-1954 possessed significant absorbance at 340 nm, the reaction rate of *Pf*GR was monitored according to GSH-mediated reduction of 5.5‘-dithiobis-(2-nitrobenzoic acid) (1.0 mM) using the value ∆ε_412_ = 27.2 mM^−1^·cm^−1^. The inhibition constants (***K***_i_) were obtained from the Cleland plots, that is, the dependence of 1/***k***_cat_ on the inhibitor concentration ([I]).

#### 4.2.2. Antiplasmodial In Vitro Activity Studies

The chloroquine-resistant *P. falciparum* strain FcB1 from Colombia, which is deposited in the Protist collection of Museum National d’Histoire Naturelle, Paris, France, was kindly provided by Dr. H.D. Heidrich (Max-Planck Institut für Biochemie, Martinsried bei München, Germany). *P. falciparum* FcB1 strain was maintained in continuous culture of human erythrocytes according to [62]. In vitro antiplasmodial activity was determined using a modification of the semiautomatic microdilution technique [63]. Stock solutions of test compounds in DMSO were serially diluted with culture medium and added to asynchronous parasite cultures (1% parasite infected cells and 1% final hematocrit) for 24 h, at 37 °C, prior to the addition of 1.825 MBq of [^3^H]-hypoxanthine (0.37–1.11 TBq/mmol), for 24 h. The growth inhibition for each compound concentration was determined according to the radioactivity incorporation into the treated culture as compared with that in the control culture. The experiments were repeated in triplicate.

#### 4.2.3. Statistical Analysis and Calculations

The octanol/water distribution coefficients at pH 7.0 (log *D*) of compounds were calculated using LogD Predictor (https://chemaxon.com). The multiparameter regression analysis was performed using Statistica (version 4.3, StatSoft, Toronto, ON, Canada).

## Figures and Tables

**Table 1:** Single-electron reduction midpoint potentials (*E*^1^_7_) of nitroaromatic compounds, their concentrations for 50% *Plasmodium falciparum* growth inhibition (IC_50_), their calculated octanol/water distribution coefficients at pH 7.0 (log *D*), and their apparent bimolecular reduction rate constants by *Plasmodium falciparum* ferredoxin:NADP^+^ oxidoreductase (*Pf*FNR) (*k*_cat_/*K*_m_).

No.	Compound	*E*^1^_7_ (V) [35]	IC_50_ (µM) [19]	log *D*	*k*_cat_/*K*_m_ (M^−1^·s^−1^)
**1**	Nitrobenzene	−0.485	473 ± 113	1.91	5.5 ± 0.8 × 10^1^
**2**	4-Nitrobenzoic acid	−0.425	360 ± 16; 450 ± 70.7 ^b^	−1.66	4.5 ± 0.6 × 10^2^
**3**	CB-1954	−0.380	48.5 ± 5.0 ^b^	0.64	2.8 ± 0.3 × 10^3^
**4**	4-Nitroacetophenone	−0.355	172 ± 8.0	1.47	3.3 ± 0.3 × 10^3^
**5**	3,5-Dinitrobenzoic acid	−0.350	390 ± 17	−1.79	3.9 ± 0.3 × 10^3^
**6**	1,3-Dinitrobenzene	−0.345	50.5 ± 2.4	1.85	2.7 ± 0.3 × 10^3^
**7**	4-Nitrobenzaldehyde	−0.325	79 ± 28	1.63	4.0 ± 0.3 × 10^3^
**8**	3,5-Dinitrobenzamide	−0.311	30.3 ± 3.1; 26.5 ± 6.4 ^b^	0.7	4.9 ± 0.4 × 10^3^
**9**	1,2-Dinitrobenzene	−0.287	11.7 ± 1.1	1.85	1.1 ± 0.2 × 10^4^
**10**	Nitrofurantoin	−0.255	12.9 ± 1.3	−0.25	6.8 ± 0.7 × 10^4^
**11**	Nifuroxime	−0.255	14.7 ± 0.8	−0.34	3.3 ± 0.4 × 10^4^
**12**	1,4-Dinitrobenzene	−0.255	0.26 ± 0.03	1.85	9.3 ± 0.8 × 10^4^
**13**	2,4,6-Trinitrotoluene	−0.253	9.4 ± 7.8 ^b^	2.31	1.3 ± 0.1 × 10^4^
**14**	*N*-Methylpicramide	−0.247	7.3 ± 1.1 ^b^	1.92	6.8 ± 0.5 × 10^4^
**15**	Nitrofuran IIIa	−0.225 ^a^	17.1 ± 1.5	0.27	4.8 ± 0.5 × 10^4^
**16**	Nitrofuran IIIb	−0.225 ^a^	4.5 ± 0.3	2.64	1.6 ± 0.2 × 10^5^
**17**	Nitrofuran IIIc	−0.225 ^a^	7.4 ± 0.3	2.87	n.d.
**18**	Nitrofuran IIId	−0.225 ^a^	7.4 ± 0.3	3.23	n.d.
**19**	Nitrofuran IIIe	−0.225 ^a^	9.2 ± 0.3	2.24	n.d.
**20**	Nitrofuran IIIf	−0.225 ^a^	11.1 ± 0.4	2.62	n.d.
**21**	Nitrofuran IIIg	−0.225 ^a^	6.4 ± 0.4	2.45	8.3 ± 0.7 × 10^4^
**22**	Nitrofuran IIIh	−0.225 ^a^	4.3 ± 0.3	2.62	n.d.
**23**	Tetryl	−0.191	4.1 ± 0.8 ^b^	1.38	2.0 ± 0.3 × 10^5^

^a^ The *E*^1^_7_ values are taken from [19], ^b^ The values of IC_50_ determined in this work. n.d., not determined.

**Table 2:** Inhibition constants of nitroaromatic compounds acting on *P. falciparum* and human erythrocyte glutathione reductases (HGRs), calculated under constant concentration of NADPH (100 μM) and varied concentration of GSSG.

No.	Compound	*K*_i_ (μM)	
		*Pf*GR ^a^	HGR ^b^
**1**	Nitrobenzene	≥6000	≥2000
**2**	4-Nitrobenzoic acid	1200 ± 180	800
**3**	CB-1954	350 ± 40	≥1000
**4**	4-Nitroacetophenone	70 ± 11	400
**5**	3,5-Dinitrobenzoic acid	220 ± 29	350
**6**	1,3-Dinitrobenzene	40 ± 5.0	320; 350 ± 30^a^
**7**	4-Nitrobenzaldehyde	25 ± 4.0	290
**8**	3,5-Dinitrobenzamide	75 ± 9.0	≥1000
**9**	1,2-Dinitrobenzene	30 ± 4.0	≥1000
**10**	Nitrofurantoin	9.0 ± 1.0	200
**11**	Nifuroxime	32 ± 5.0	200
**12**	1,4-Dinitrobenzene	0.85 ± 0.13	71
**13**	2,4,6-Trinitrotoluene	8.0 ± 2.0	6.0 ^c^; 5.2 ± 0.6 ^a^
**14**	*N*-Methylpicramide	5.9 ± 0.6	10 ^c^
**15**	Nitrofuran IIIa	9.0 ± 1.0	3.0; 3.5 ± 0.2 ^a^
**16**	Nitrofuran IIIb	25 ± 3.0	2.5
**17**	Nitrofuran IIIc	115 ± 17	25
**18**	Nitrofuran IIId	50 ± 6.0	≥300
**19**	Nitrofuran IIIe	5.0 ± 1.0	2.5
**20**	Nitrofuran IIIf	75 ± 10	42.5
**21**	Nitrofuran IIIg	35 ± 5.0	25
**22**	Nitrofuran IIIh	100 ± 12	45
**23**	Tetryl	2.3 ± 0.5	14 ^c^

^a^*K*_i_ determined in present work, ^b^ taken from [19], ^c^ taken from [42].

**Table 3:** Kinetic characterization of nitro- and quinone reductase reactions of *Plasmodium falciparum* flavoenzymes.

Enzyme	Oxidants	
	Nitrofurans, Nitrobenzenes	Menadione
	*E*^1^_7_ = −0.25–−0.19 V	
*Pf*FNR	*k*_cat_ > 20 s^−1^, *k*_cat_/*K*_m_ = 4.8 × 10^4^ – 1.6×10^5^ M^−1^·s^−1^, this work	*k*_cat_ = 14 s^−1^, *k*_cat_/*K*_m_ = 1.0 ×10^6^ M^−1^·s^−1^ [30]
*Pf*GR	*k*_cat_ = 0.06–5.9 s^−1^, *k*_cat_/*K*_m_ = 7.6 × 10^3^ – 110 M^−1^·s^−1^, this work	*k*_cat_ = 0.16 s^−1^, *k*_cat_/*K*_m_ = 2.0 × 10^3^ M^−1^·s^−1^ [54]
*P. falciparum* thioredoxin reductase		*k*_cat_ = 31 s^−1^, *k*_cat_/*K*_m_ = 1.6 × 10^5^ M^−1^·s^−1^ [54]
*P. falciparum* type II NADH dehydrogenase		*k*_cat_ = 0.1 s^−1^ [55]

## Supplementary Materials

The following are available online. Figure S1: Dependence of activity of nitroaromatic compounds against *P. falciparum* FcB1(IC_50_) on the values of their single-electron reduction midpoint potential (*E*^1^_7_), Figure S2: Dependence of activity of nitroaromatic compounds against *P. falciparum* FcB1(IC_50_) on the values of their log *D*, Figure S3: Dependence of activity of nitroaromatic compounds against *P. falciparum* FcB1(IC_50_) on the values of their inhibition constant (*K*_i_) of *P. falciparum* glutathione reductase.

## Abbreviations

ArNO_2_	Nitroaromatic compound
*E* ^1^_7_	Single-electron reduction midpoint potential of nitroaromatic compound (redox potential of ArNO_2_/ArNO_2_^−.^ couple) at pH 7.0
HGR	Human erythrocyte glutathione reductase
IC_50_	Compound concentration causing 50% parasite growth inhibition
*k*_cat_	Enzyme catalytic constant
*k*_cat_/*K*_m_	Enzyme bimolecular rate constant (catalytic efficiency)
*K*_i_	Enzyme inhibition constant
log *D*	Octanol/water distribution coefficient at pH 7.0
*Pf*FNR	*P. falciparum* ferredoxin:NADP^+^ oxidoreductase
*Pf*GR	*P. falciparum* glutathione reductase
ROS	Reactive oxygen species
TNT	2,4,6-Trinitrotoluene
TR	Trypanothione reductase

## References

[1] Bhatt S., Weiss D.J., Cameron E., Bisancia D., Mappin B., Dalrymple U., Battle K., Moyes C.L., Henry A., Eckhoff P.A. (2015). The effect of malaria control on *Plasmodium falciparum* in Africa betwen 2000 and 2015. Nature.

[2] Müller S. (2004). Redox and antioxidant systems of the malaria parasite *Plasmodium falciparum*. Mol. Microbiol..

[3] Pelfrene E., Harvey Allchurch M., Ntamabyaliro N., Nambasa A., Ventura F.V., Nagercoil N., Cavaleri M. (2019). The European Medicines Agency’s scientific opinion on oral feximidazole for human African trypanosomiasis. PLoS Negl. Trop. Dis..

[4] Guissani A., Henry Y., Lougmani N., Hickel B. (1990). Kinetic studies of four types of nitroheterocyclic radicals by pulse radiolysis. Correlation of pharmacological properties to decay rates. Free Radic. Biol. Med..

[5] Wilkinson S.R., Bot C., Kelly J.M., Hall B.S. (2011). Trypanocidal activity of nitroaromatic prodrugs: Current treatments and future perspectives. Curr. Top. Med. Chem..

[6] Pal C., Bandyopadhyay H. (2012). Redox active antiparasitic drugs. Antiox. Redox Signal..

[7] Bironaitė D.A., Čėnas N.K., Kulys J.J. (1991). The rotenone-insensitive reduction of quinones and nitrocompounds by mitochondrial NADH-ubiquinone reductase. Biochim. Biophys. Acta.

[8] Čėnas N., Nemeikaitė-Čėnienė A., Sergedienė E., Nivinskas H., Anusevičius Ž., Šarlauskas J. (2001). Quantitative structure-activity relationships in enzymatic single-electron reduction of nitroaromatic explosives: Implications for their cytotoxicity. Biochim. Biophys. Acta.

[9] Anusevičius Ž., Nivinskas H., Šarlauskas J., Sari M.-A., Boucher J.-L., Čėnas N. (2013). Single-electron reduction of quinone and nitroaromatic xenobiotics by recombinant rat neuronal nitric oxide synthase. Acta Biochim. Pol..

[10] Moussaoui M., Misevičienė L., Anusevičius Ž., Marozienė A., Lederer F., Baciou L., Čėnas N. (2018). Quinones and nitroaromatic compounds as subversive substrates of *Staphylococcus aureus* flavohemoglobin. Free Rad. Biol. Med..

[11] Henderson G.B., Ulrich P., Fairlamb A.H., Rosenberg I., Pereira M., Sela I., Cerami A. (1988). “Subversive” substrates for the enzyme trypanothione disulfide reductase: Alternative approach to chemotherapy of Chagas disease. Proc. Natl. Acad. Sci. USA.

[12] Čėnas N., Bironaitė D., Dičkancaitė E., Anusevičius Ž., Šarlauskas J., Blanchard J.S. (1994). Chinifur, a selective inhibitor and subversive substrate for *Trypanosoma congolense* trypanothione reductase. Biochem. Biophys. Res. Commun..

[13] Millet R., Maes L., Landry V., Sergheraert C., Davioud-Charvet E. (2002). Antitrypanosomal activities and cytotoxicity of 5-nitro-2-furancarbohydrazides. Bioorg. Med. Chem. Lett..

[14] Arias D.G., Herrera F.E., Garay A.S., Rodrigues D., Forastieri P.S., Luna L.E., Bürgi M.D., Prieto A.A., Iglesias A.A., Cravero R.M. (2017). Rational design of nitrofuran derivatives: Synthesis and valuation as inhibitors of *Trypanosoma cruzi* trypanothione reductase. Eur. J. Med. Chem..

[15] Leroux A.E., Krauth-Siegel R.L. (2016). Thiol redox biology of trypanosomatids as potential targets for chemotherapy. Mol. Biochem. Parasitol..

[16] Ilari A., Genovese I., Fiorillo F., Battista T., De Iona I., Fiorello A., Colotti G. (2018). Toward a drug against all kinetoplastids: From LeishBox to specific and potent trypanothione reductase inhibitors. Mol. Pharm..

[17] Kuntz A.N., Davioud-Charvet E., Dessolin J., Sayed A.A., Califf L.L., Arnér E.S.J., Williams D.L. (2007). Thioredoxin glutathione reductase from Schistosoma mansoni: An essential parasite enzyme and a key drug target. PLoS Med..

[18] Li T., Ziniel P.D., He P.Q., Kommer V.P., Crowther G.J., He M., Lin Q., Van Voorhis W.C., Williams D.L., Wang M.W. (2015). High-throughput screening against thioredoxin glutathione reductase identifies novel inhibitors with potential therapeutic value for schistosomiasis. Infect. Dis. Poverty.

[19] Grellier P., Šarlauskas J., Anusevičius Ž., Marozienė A., Houeee-Levin C., Schrevel J., Čėnas N. (2001). Antiplasmodial activity of nitroaromatic and quinoidal compounds: Redox potential vs inhibition of erythrocyte glutathione reductase. Arch. Biochem. Biophys..

[20] Wiesner J., Kettler K., Sakowski J., Ortmann R., Jomaa H., Schlitzer M. (2003). Structure-activity relationships of novel anti-malarial agents: Part 5. *N*-(4-acylamino-3-benzoylphenyl)-[5-(4-nitrophenyl)-2-furyl]acrylic acid amides. Bioorg. Med. Chem. Lett..

[21] Tukulula M., Sharma R.-K., Meurillon M., Mahajan A., Naran K., Warner D., Huang J., Mekonnen B., Chibale K. (2012). Synthesis and antiplasmodial and antimycobacterial evaluation of new nitroimidazole and nitroimidazooxazine derivatives. ACS Med. Chem. Lett..

[22] Munigunti R., Gathiaka S., Acevedo O., Sahu R., Tekwari B., Calderon A.I. (2013). Characterization of PfTrxR inhibitors using antimalarial assays and in silico techniques. Chem. Cent. J..

[23] Burkard L., Scheuermann A., Simithy J., Calderon A.I. (2015). Development of a functional assay to detect inhibitors of *Plasmodium falciparum* glutathione reductase utilizing liquid chromatography-mass spectrometry. Biomed. Chromatogr..

[24] Vennerstrom L.J., Eaton J.W. (1988). Oxidants, oxidant drugs, and malaria. J. Med. Chem..

[25] Čėnas N.K., Bironaitė D.A., Kulys J.J., Sukhova N.M. (1991). Interaction of nitrofurans with glutathione reductase. Biochim. Biophys. Acta.

[26] Cakmak R., Durdagi S., Ekinci D., Sentürk M., Topal G. (2011). Design, synthesis and biological evaluation of novel nitroaromatic compounds as potent glutathione reductase inhibitors. Bioorg. Med. Chem. Lett..

[27] Sarma G.N., Savvides S.N., Becker K., Schirmer M., Schirmer R.H., Karplus P.A. (2003). Glutathione reductase of the malarial parasite *Plasmodium falciparum*: Crystal structure and inhibitor development. J. Mol. Biol..

[28] Tyagi C., Bathke J., Goyal S., Fischer M., Dahse H.M., Chacko S., Becker K., Grover A. (2015). Targeting the intersubunit cavity of *Plasmodium falciparum* glutathione reductase by a novel natural inhibitor: Computational and experimental evidence. Int. J. Biochem. Cell Biol..

[29] Böhme C.C., Arscott D., Becker K., Schirmer R.H., Williams C.H. (2000). Kinetic characterization of glutathione reductase from the malarial parasite *Plasmodium falciparum*. Comparison with the human enzyme. J. Biol. Chem..

[30] Grellier P., Marozienė A., Nivinskas H., Šarlauskas J., Aliverti A., Čėnas N. (2010). Antiplasmodial activity of quinones: Roles of aziridinyl substituents and the inhibition of *Plasmodium falciparum* glutathione reductase. Arch. Biochem. Biophys..

[31] O’Brien P.J., Wong W.C., Silva J., Khan S. (1990). Toxicity of nitrobenzene compounds towards isolated hepatocytes: Dependence on reduction potential. Xenobiotica.

[32] Lukevits E., Demicheva L. (1993). Biological activity of furan derivatives. Chem. Heterocycl. Comp. (Riga).

[33] Daghastanli N.A., Degterev L.A., Tedesco A.C., Borisevitch I.E. (2004). Phototoxicity of a 5-nitrofuran-ethenyl-quinoline antiseptic (Quinifuryl) to P388 mouse leukemia cells. Braz. J. Med. Biol. Res..

[34] Tseng C.H., Tzeng C.C., Chiu C.C., Hsu C.Y., Chou C.-K., Chen Y.L. (2015). Discovery of 2-[2-(5-nitrofuran-2-yl) vinyl]quinoline derivatives as a novel type of antimetastatic agents. Bioorg. Med. Chem..

[35] Wardman P. (1989). Reduction potentials of one-electron couples involving free radicals in aqueous solution. J. Phys. Chem. Ref. Data.

[36] Balconi E., Pennati A., Crobu D., Pandini V., Cerutti R., Zanetti G., Aliverti A. (2009). The ferreroxin-NADP^+^ reductase/ferredoxin electron transfer system of *Plasmodium falciparum*. FEBS J..

[37] Seeber F., Aliverti A., Zanetti G. (2005). The plant-type ferredoxin-NADP^+^ reductase/ferredoxin redox system as a possible drug target against apicomplexan human parasites. Current Pharm. Des..

[38] Zhang M., Wang C., Otto T.D., Oberstaller J., Liao X., Adapa S.R., Udenze K., Bronner I.F., Casandra D., Mayho M. (2018). Uncovering the essential genes of the human parasite *Plasmodium falciparum* by saturation mutagenesis. Science.

[39] Bironaite D.A., Chenas N.K., Kulis Y.Y. (1992). Nonphysiological redox agents are reduced in the NADP(H) binding-site of glutathione reductase. Biochemistry-Moscow.

[40] Belorgey D., Lanfranchi D.A., Davioud-Charvet E. (2013). 1,4-Naphthoquinones and other NADPH- dependent glutathione reductase-catalyzed redox cyclers as antimalarial agents. Curr. Pharm. Des..

[41] Salmon-Chemin L., Lemaire A., De Freitas S., Deprez B., Sergheraert C.E., Davioud-Charvet E. (2000). Parallel synthesis of a library of 1,4-naphthoquinones and automatic screening of potential inhibitors of trypanothione reductase from *Trypanosoma cruzi*. Bioorg. Med. Chem. Lett..

[42] Miškinienė V., Anusevičius Ž., Marozienė A., Kliukienė R., Nivinskas H., Šarlauskas J., Čėnas N., Becker K., Ghisla S., Kroneck P., Macheroux P., Sund H. (1999). Tetryl as inhibitor and subversive substrate for human erythrocyte glutathione reductase. Proceedings of the 13th International Symposium on Flavins and Flavoproteins.

[43] Foley M., Tilley L. (1998). Quinoline antimalarials: Mechanisms of action and resistance and prospects for new agents. Pharmacol. Ther..

[44] Famin O., Krugliak M., Ginsburg H. (1999). Kinetics of inhibition of glutathione-mediated degradation of protoporphyrin IX by antimalarial drugs. Biochem. Pharmacol..

[45] Marva E., Chevion M., Golenser J. (1991). The effects of free radicals induced by paraquat and copper on the in vitro development of Plasmodium falciparum. Free Rad. Res. Comun..

[46] Chaudhari R., Sharma S., Patankar S. (2017). Glutathione and thioredoxin systems of the malaria parasite *Plasmodium falciparum: Partners* in crime?. Biochem. Biophys. Res. Commun..

[47] Ayi K., Cappadoro M., Branca M., Turrini F., Arese P. (1998). *Plasmodium falciparum* glutathione metabolism and growth are independent of glutathione system of host erythrocyte. FEBS Lett..

[48] González-Párraga P., Hernández A., Argüelles J.C. (2003). Role of antioxidant enzymatic defences against oxidative stress, H_2_O_2_, and the acquisition of oxidative tolerance in *Candida albicans*. Yeast.

[49] Dixit V., Pandey V., Shyam R. (2001). Differential antioxidative responses to cadmium in roots and leaves of pea (*Pisum sativum* L. cv. *Azad*). J. Exp. Bot..

[50] Pedrosa R.C., De Bem A.F., Locatelli C., Pedrosa R.C., Geremias R., Wilhelm Filho D. (2001). Time-dependent oxidative stress caused by benznidazole. Redox Rep..

[51] Čėnas N., Prast S., Nivinskas H., Šarlauskas J., Arner E.S.J. (2006). Interaction of nitroaromatic compounds with the mammalian selenoprotein thioredoxin reductase and the relation to induction of apoptosis in human cancer cells. J. Biol. Chem..

[52] Monti D., Basilico N., Parapini S., Pasini E., Olliaro P., Taramelli D. (2002). Does chloroquine really act through oxidative stress?. FEBS Lett..

[53] Becker K., Tilley L., Vennerstrom J.L., Roberts D., Rogerson S., Ginsburg H. (2004). Oxidative stress in malaria parasite-infected erythrocytes: Host-parasite interactions. Int. J. Parasitol..

[54] Morin C., Besset T., Moutet J.-C., Fayolle M., Brückner M., Limosin D., Becker K., Davioud-Charvet E. (2008). The aza-analogues of 1,4-naphthoquinones are potent substrates and inhibitors of plasmodial thioredoxin and glutathione reductases and of human erythrocyte glutathione reductase. Org. Biomol. Chem..

[55] Dong C.K., Patel V., Yang J.C., Dvorin J.D., Duraisingh M.T., Clardy J., Wirth D.F. (2009). Type II NADH dehydrogenase of the respiratory chain of *Plasmodium falciparum* and its inhibitors. Bioorg. Med. Chem. Lett..

[56] Čėnas N.K., Arscott D., Williams C.H., Blanchard J.S. (1994). Mechanism of reduction of quinones by *Trypanosoma congolense* trypanothione reductase. Biochemistry.

[57] Kröger M., Fels G. (2000). ^14^C-TNT synthesis revisited. J. Label. Compd. Radiopharm..

[58] Hughes E.D., Ingold C., Pearson R.B. (1958). Nitration at nitrogen and oxygen centers. Part I. Kinetics and conversion of secondary amines into nitroamines. J. Chem. Soc..

[59] Khan A.H., Ross W.C.J. (1969). Tumor-growth inhibitory nitrophenylaziridines and related compounds: Structure-activity relationships. Chem. Biol. Interact..

[60] Sukhova N.M., Lidaka M.J., Voronova V.A., Zidermane A.A., Kravchenko I.M., Dauvarte A.Z., Preisa I.E., Meirena I.E. (1980). Substituted 2-[2′-(5”-nitrofuryl-2”)vinyl- and 4′-(5”-nitrofuryl-2”)-1,3- butadienyl)-quinoline-4-carboxylic acid amides and salts thereof. U.S. Patent.

[61] Lukevits E., Lapina T.V., Sukhova N.M., Zidermane A.A., Dauvarte A.Z., Voronova V.A. (1981). Nitrogen-containing organosilicon compounds. CIV. Synthesis and antiblastic activity of amides of quinoline carboxylic acids. Pharm. Chem. J..

[62] Trager W., Jensen J.B. (1976). Human malaria parasites in continuous culture. Science.

[63] Desjardins R.E., Canfield C.J., Haynes J.D., Chullay J.D. (1979). Quantitative assessment of antimalarial activity in vitro by semiautomated microdilution technique. Antimicrob. Agents Chemother..

