# Supplementary material for: Antiplasmodial Activity of Nitroaromatic Compounds: Correlation with Their Reduction Potential and Inhibitory Action on Plasmodium falciparum Glutathione Reductase
Source: Molecules. 2019 Dec 10;24(24):4509. doi: 10.3390/molecules24244509 (PMC6943496; doi:10.3390/molecules24244509)
Supplement: Supplementary file 1 [file molecules-24-04509-s001.pdf]

molecules

MDPI

Supplementary Materials

Antiplasmodial Activity of Nitroaromatic Compounds: Correlation with Their Reduction Potential and Inhibitory Action on *Plasmodium falciparum* Glutathione Reductase

Audronė Marozienė [1], Mindaugas Lesanavičius [1], Elisabeth Davioud-Charvet [2], Alessandro Aliverti [3], Philippe Grellier [4], Jonas Šarlauskas [1], and Narimantas Čėnas [1,*]

[1] Department of Xenobiotics Biochemistry, Institute of Biochemistry of Vilnius University, Saulėtekio 7, LT-10257 Vilnius, Lithuania; audrone.maroziene@bchi.vu.lt (A.M.); mindaugas.lesanavicius@gmail.com (M.L.); jonas.sarlauskas@bchi.vu.lt (J.Š.)

[2] UMR7042 CNRS-Unistra-UHA, Laboratoire d'Innovation Moléculaire et Applications (LIMA), Bioorganic and Medicinal Chemistry Team, European School of Chemistry, Polymers and Materials, 25 rue Becquerel, F-67087 Strasbourg, France; elisabeth.davioud@unistra.fr

[3] Department of Biosciences, Universita degli Studi di Milano, via Celoria 26, I-20133 Milano, Italy; alessandro.aliverti@unimi.it

[4] MCAM, UMR7245, Museum National d'Histoire Naturelle, CNRS, 61 rue Buffon, F-75231 Paris CEDEX 05, France; philippe.grellier@mnhn.fr

* Correspondence: narimantas.cenas@bchi.vu.lt; Tel.: +370-5-223-4392

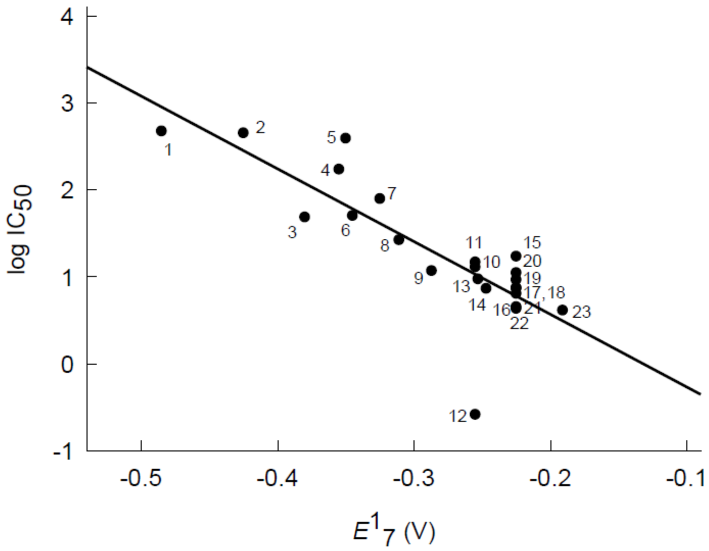

Figure S1. Dependence of activity of nitroaromatic compounds against *P. falciparum* FcB1 (IC_{50}) on the values of their single-electron reduction midpoint potential ($E^1{}_7$). The numbers of compounds and data are taken from Table 1.

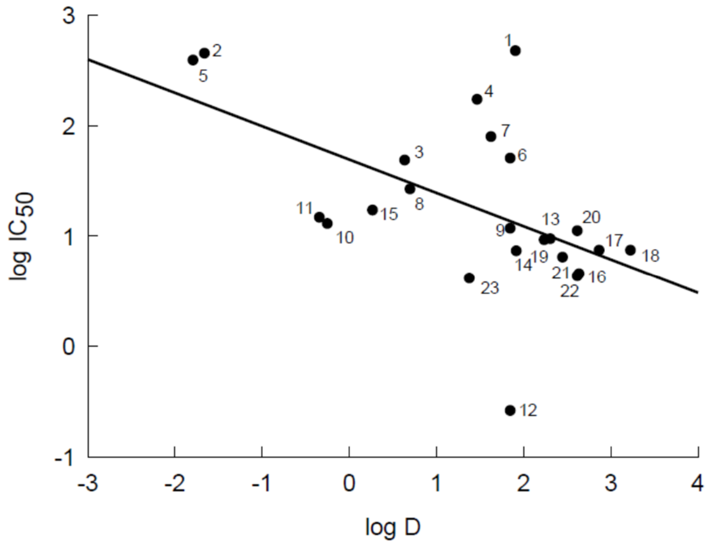

Figure S2. Dependence of activity of nitroaromatic compounds against *P. falciparum* FcB1 (IC_{50}) on the values of their log *D*. The numbers of compounds and data are taken from Table 1.

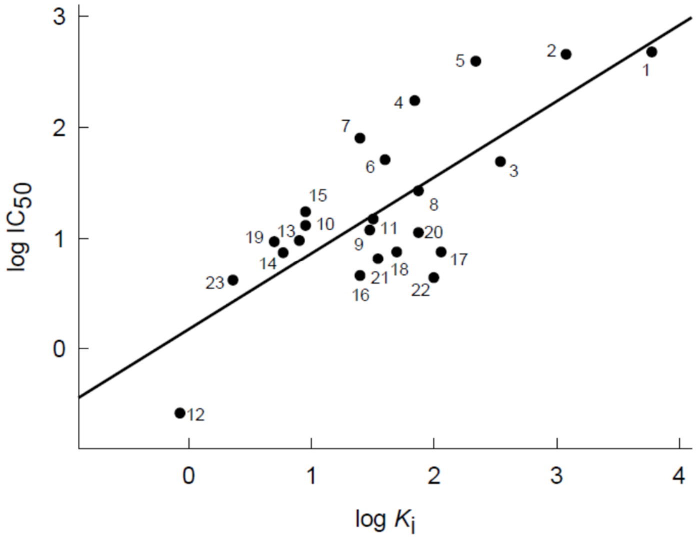

Figure S3. Dependence of activity of nitroaromatic compounds against *P. falciparum* FcB1 (IC_{50}) on the values of their inhibition constant (K_i) of *P. falciparum* glutathione reductase. The numbers of compounds and data are taken from Table 2.
